# Red and White

**Published:** 1887-07

**Authors:** 


### RED AND WHITE.

The rose unto the lily said,
When all its pearly leaves were spread
To the warm sunshine overhead ;
  Prithee sweet sister, why so pale
    And *I* so glowing red ?
The lily graciously replied,
*My* home is on the yielding tide
  And *yours*, the grassy vale ;
*You* taste the drops by heaven diffused
O'er the glad earth, but *I* am used
To founts that 'neath the waters lie,
And all my simple wants supply :—
The glory of the morn is *mine !*
His magic touch of silvery light
Awakes to life my petals white,
  And folds them with the day's decline ;
But *you*, that wear the blush of e'en,
Beneath the starlight's golden sheen,
Give equal welcome to the night.
*I* love the waves the breezes woo,
The dimpling waves ; they love *me* too,
And lap me as a mother, *you*
Within your leafy bower so green,
Hold regal court, a very queen.
Thus nature, ever kind, contrives
For every living thing a way ;
And be our station what it may,
*We take our color from our lives !*

